# COVID-19 Testing of United States-Bound Agricultural Workers in Mexico

**DOI:** 10.1007/s10903-023-01517-x

**Published:** 2023-08-28

**Authors:** Jessica Teleaga, Zachary A. White, Joaquin Cervantes, Roberto Assael, Gerardo Barrera, Sean Toney, Nina Marano, Alfonso Rodriguez Lainz, Chantal Assael, Alexandra Ortega, Courtney G. Chappelle, Nirma Bustamante, Kathleen Moser, Drew L. Posey

**Affiliations:** 1 Division of Global Migration and Quarantine, Centers for Disease Control and Prevention, 1600 Clifton Rd, NE MS H16-4, 30333 Atlanta, GA Georgia; 2 Oak Ridge Institute for Science and Education Fellowship Program, Oak Ridge Associated Universities, P.O. Box 117, Oak Ridge, TN 37831-0117 USA; 3 Clínica Médica Internacional, Avenida Ramón Rivera Lara, 9020 Fraccionamiento Las Lunas, C.P. 32543 Ciudad Juárez, Chihuahua Mexico; 4 International Panel Physicians Association, 118 Mesa Park Drive, Suite 100, El Paso, TX 79912 USA

**Keywords:** COVID-19, H-2A visa, Agricultural workers, Migration, Testing

## Abstract

The COVID-19 pandemic presents global health, welfare, and economic concerns. The agricultural workforce has experienced adverse effects, placing the U.S. food supply at risk. Agricultural workers temporarily travel to the United States on H-2A visas to supplement the agricultural workforce. Approximately 300,000 agricultural workers enter the United States with H-2A visas each year; over 90.0% are from Mexico. During February–May 2021, a COVID-19 testing pilot was performed with Clínica Médica Internacional (CMI), a clinic that performs medical examinations for US-bound immigrants, to determine the SARS-CoV-2 infection status of H-2A agricultural workers in Mexico before entry to the US. The CerTest VIASURE Real Time PCR Detection Kit was used. Participants’ demographic information, test results, and testing turnaround times were collected. Workers who tested positive for SARS-CoV-2 completed isolation before US entry. During the pilot, 1195 H-2A workers were tested; 15 (1.3%) tested positive. Average reporting time was 31 h after specimen collection. This pilot demonstrated there is interest from H-2A employers and agents in testing the H-2A community before US entry. Testing for SARS-CoV-2 can yield public health benefit, is feasible, and does not delay entry of temporary agricultural workers to the US.

## Introduction

The COVID-19 pandemic presented many challenges for migrant farmworkers traveling to the United States and the farms where they work. One important source of farmworkers in the United States is the H-2A visa program. This program allows US employers or agents who meet specific regulatory requirements to bring foreign nationals to the United States to fill temporary agricultural jobs. A U.S. Citizenship and Immigration Services (USCIS) Form I-129, Petition for a Nonimmigrant Worker must be filed on behalf of a prospective worker by a US employer, a US agent as described in the regulations, or an association of US agricultural producers named as a joint employer [1, 2]. The number of H-2A visas issued by the Department of State has quadrupled since 2007 (50,791) and continues to increase [3, 4]. Of the 213,394 H-2A visas issued in 2020, 198,000 (93.0%) were issued to Mexican nationals [3].

As opposed to persons applying for an immigrant visa or applying to be admitted as a refugee, nonimmigrant visa applicants, including H-2A applicants, are not routinely required to have a medical examination before visa issuance. In addition, the CDC order issued in January 2021 requiring travelers to provide proof of negative COVID-19 test before entry to the United States applies to air travel only, and not to travelers entering the US at land ports of entry (POEs) [5]. Historically, persons issued H-2A visas in Monterrey and Tijuana, Mexico, typically enter the US at land POEs.

We sought to examine the prevalence of COVID-19 in H-2A workers before entering the United States and determine whether H-2A workers would volunteer to be tested in Mexico. CDC conducted a pilot testing program in Mexico to offer free, voluntary COVID-19 testing to H-2A workers, independent of the visa process, before entering the United States.

## Methods

CDC designed the pilot program (February–May 2021) as a cross-sectional evaluation, with a convenience sample of 1195 H-2A workers from Mexico, to investigate the prevalence of COVID-19 infection among workers before admission to the US for agricultural employment.

CDC collaborated with the National Council of Agricultural Employers (NCAE; a national association representing agricultural employers) and the US Consulate Generals in Monterrey and Tijuana to advertise the COVID-19 testing pilot among H-2A workers in Mexico [6]. CDC held multiple meetings to provide details of the pilot’s testing service. In turn, NCAE advertised the pilot through their channels to agricultural employers and agents. This H-2A worker public health response testing pilot was carried out by the International Panel Physicians Association (IPPA), with funding from the CDC. IPPA provides training and education for US panel physicians. The CerTest VIASURE Real Time PCR Detection Kit (CerTest Biotec S.L., Zaragoza, Spain) for SARS-CoV-2 was used at the two testing locations [7]. Agricultural employers or agents contacted the IPPA to schedule testing appointments on behalf of their H-2A workers. IPPA scheduled individuals on a first-come, first-served basis since testing was limited and relayed appointment schedules to Clínica Médica Internacional (CMI). For this pilot, IPPA scheduled the tests and reimbursed CMI for the cost of testing the H-2A workers who participated.

Panel physicians are local physicians designated by the U.S. Department of State to perform required medical examinations for US-bound immigrants and refugees. These examinations are not required for H-2A workers since H-2A visas are a nonimmigrant visa class. CMI, a Mexican panel physician site, based in Ciudad Juarez, co-manages a clinic in Mexico City with Servicios Medicos de la Frontera, another US panel physician site. During the COVID-19 pandemic, CMI opened COVID-19 testing sites in Monterrey and Tijuana. Of the four available CMI sites, only two were accessed for testing H-2A workers: Monterrey and Tijuana.

The CDC investigator team developed a data collection spreadsheet for those tested in the pilot. Data was collected and compiled in a CMI internal database from which variables were extracted and reported to CDC on a weekly basis. The dataset included demographic information (age, gender, and state of residence), primary outcome data including turnaround time of testing, COVID-19 test result, housing arrangements for isolation purposes, and employment visa status. Personally identifiable information on individual H-2A workers was not transmitted to CDC. Data analysis was done by using IBM SPSS 25 (IMB Corp., Armonk, New York).

This project was reviewed by the CDC and deemed not to be research; it was conducted consistent with applicable federal law and CDC policy.

## Results

Before traveling to the United States, 1195 H-2A workers were screened in Mexico. At the time of testing, only 1.4% of the workers reported already having their employment visas. Males composed 98.3% of the sample with a mean age of 30 years, ranging from 17 to 68 years (Table 1). One-third of the sample was between 17 and 25 years; 10 (0.8%) individuals refused to disclose their age.

**Table 1:** H-2A Participants’ Demographic Characteristics, Age and Sex

	Frequency	Percent (%)
Age Groups		
17–25 years old	396	33.1
26–30 years old	313	26.2
31–35 years old	192	16.1
36–40 years old	139	11.6
41–45 years old	71	5.9
46 and older	74	6.2
Unknown/Refused	10	0.8
Sex		
Male	1175	98.3
Female	20	1.7
Total	1195	100.0

**Fig. 1:**
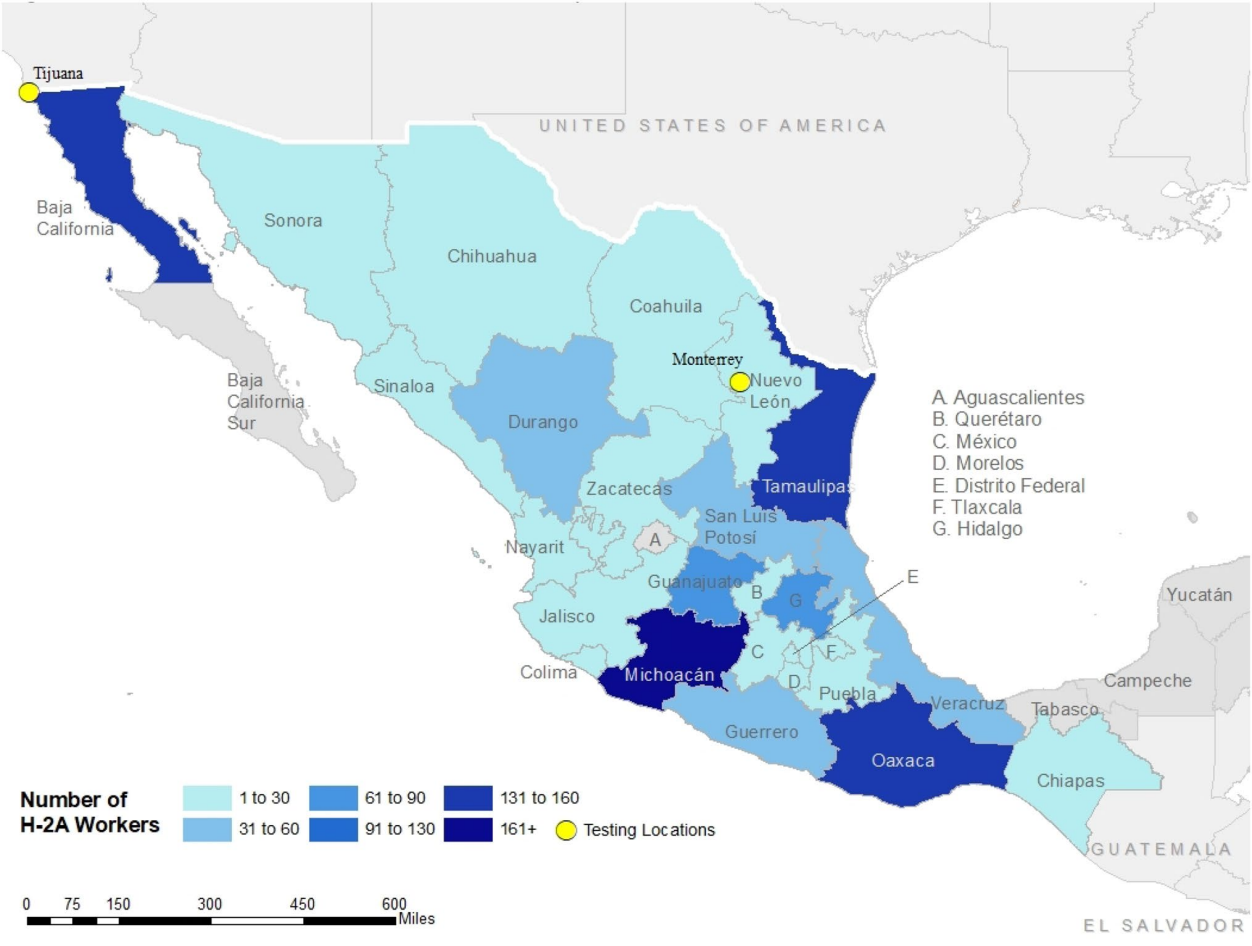
Number of H-2A Workers Tested by State of Residence

Workers came from geographically dispersed areas, with the highest concentration of H-2A workers coming from southeast Mexico (Fig. 1). The highest number of H-2A workers (N = 167) originated from Michoacán, located in the western region of Mexico.

**Fig. 2:**
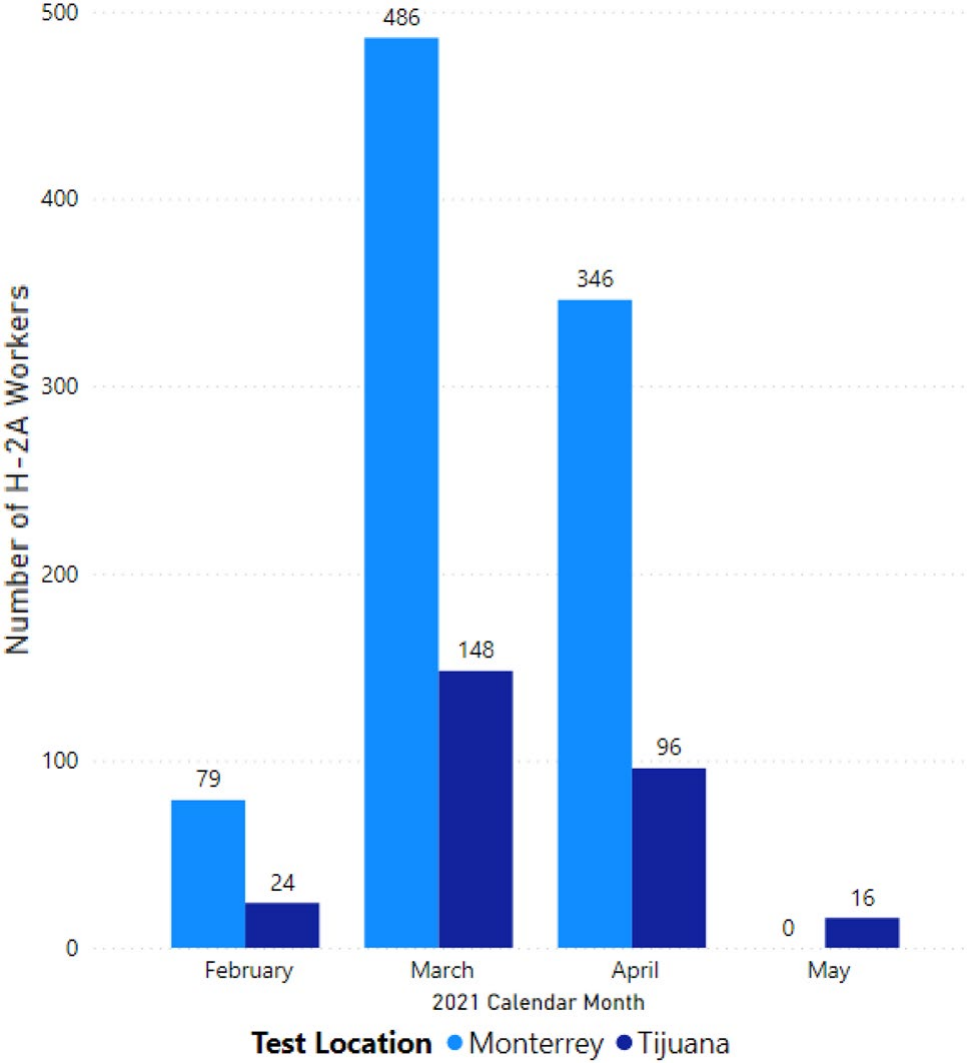
Number of H-2A Workers Tested by Location and Month

Most participants (N = 911, 76.2%) were tested at the Monterrey site, and the remaining participants (N = 284, 23.8%) were tested at the Tijuana site. The participants who volunteered for testing represented seven different US employers and one agent (Table 2). The number of workers enrolled by an employer or agent ranged from 12 to 139 H-2A workers per employer. The agent enrolled over three-fourths of the sample, 911 H-2A workers (76.2%), all were tested in Monterrey. We did not collect data on the number of employers the agent worked with. Both locations saw a peak in H-2A workers enrolled for screening during the month of March (Fig. 2).

**Table 2:** Number of H-2A Workers Represented by US Employers and Agent

	Frequency	Percent (%)
Employer A	14	1.2
Employer B	18	1.5
Employer C	24	2.0
Employer D	62	5.2
Employer E	15	1.3
Employer F	12	1.0
Employer G	139	11.6
Agent 1	911	76.2

**Fig. 3:**
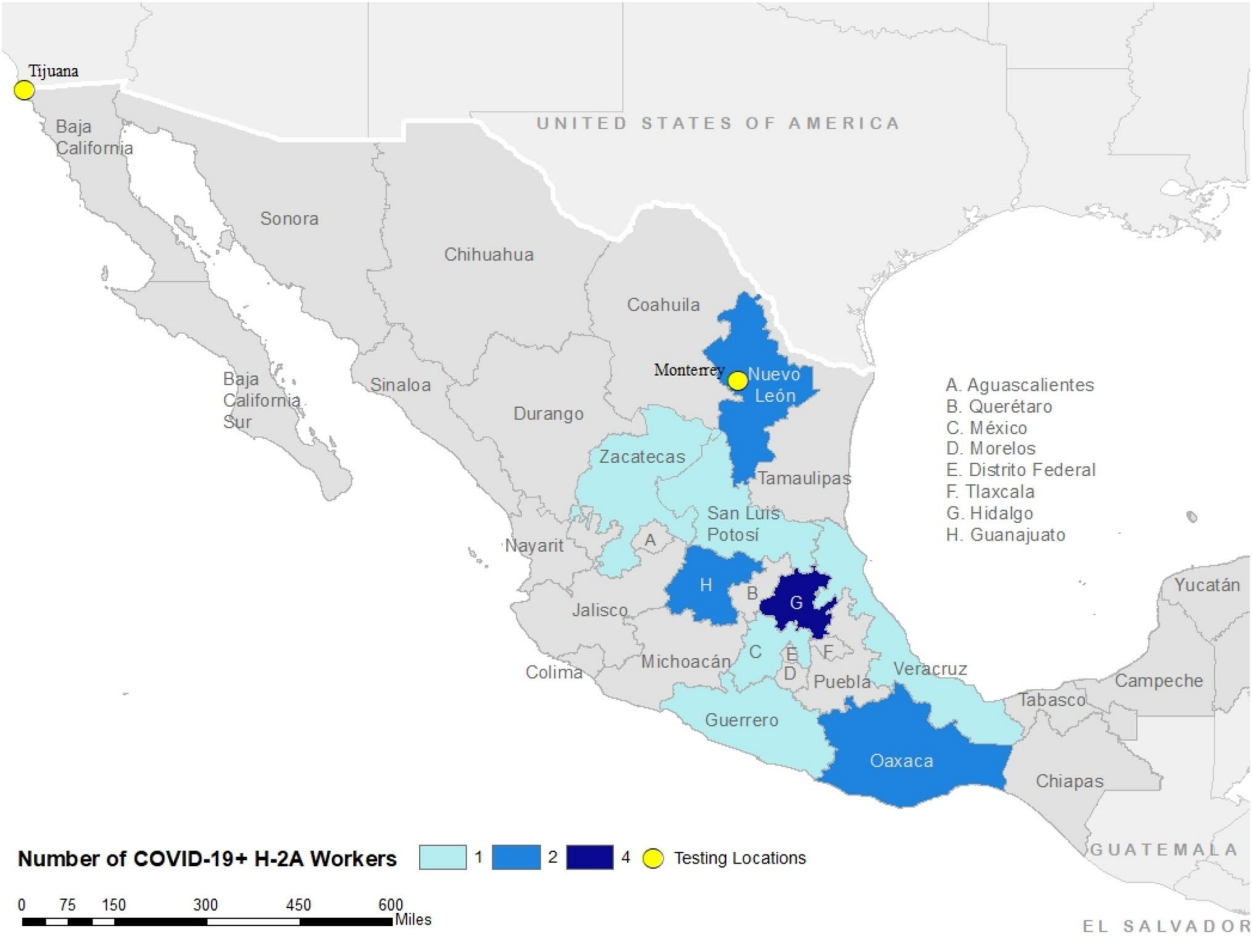
Number of H-2A COVID-19 Positive Cases by State of Residence

Overall, fifteen H-2A workers tested positive for COVID-19 (1.3%). Of those positive cases, the majority were ages 35 years or younger (60.0%), with a range in age of 22 to 59 years (mean age = 34); those between the ages of 31–35 years represented the highest number of positive cases (N = 4, 26.7%). The positive cases resided in the eastern region of Mexico, which includes Hidalgo, the state with the highest concentration of positive cases (N = 4) (Fig. 3).

Among all tested individuals, 61.9% reported that their employers would be responsible for housing them for isolation in Mexico if their test result was positive. Conversely 38.1% reported they would be responsible for their isolation accommodations following a positive test result. Among the positive cases, 53.3% reported their employers assumed the responsibility of arranging isolation accommodations.

Three markers were used to calculate turnaround times: time of sample collection, test completion, and result reported. Turnaround times were calculated from sample collection to test completion (mean = 21.98 h), test completion to result reported (mean = 9.09 h), and sample collection to result reported (mean = 31.32 h). On average, results were reported the day after sample collection. When stratified by testing location, Monterrey had longer times from test completion to result reported (mean = 10.24 h) and sample collection to result reported (mean = 32.25 h) (Table 3).

**Table 3:** Turnaround Times of Laboratory Test

	Total (N)	Minimum (h)	Maximum (h)	Mean (h)	Std. deviation
Overall turnaround times					
Sample collection—test completion	1195	4	43	21.98	12.203
Test completion—result reported	1195	0	24	9.09	6.768
Sample collection—result reported	1195	6	59	31.32	16.464
Turnaround times by testing location					
Monterrey					
Sample collection—test completion	911	6	43	21.75	12.272
Test completion—result reported	911	0	24	10.24	7.092
Sample collection—result reported	911	7	59	32.25	17.355
Tijuana					
Sample collection—test completion	284	4	32	22.73	11.971
Test completion—result reported	284	1	16	5.41	3.697
Sample collection—result reported	284	6	38	28.33	12.782

The H-2A workers who disclosed their intended US locations reported California, Florida, North Carolina, South Carolina, Virginia, or Oregon as their first US work location with the most common reporting South Carolina (47.3%) (Table 4). Of the fifteen positive cases, 11 (73.3%) reported South Carolina, and one (6.7%) reported Florida as their first intended US location. Three (20.0%) of the fifteen positive cases did not report an intended US location.

**Table 4:** First Intended US Location for All H-2A Workers Tested

	Frequency	Percent (%)
South Carolina	565	47.3
California	239	20.0
Florida	193	16.2
Oregon	45	3.8
North Carolina	35	2.9
Virginia	32	2.7
Unknown	86	7.2
Total	1195	100.0

## Discussion

To our knowledge, this CDC pilot is the first project to test US-bound H-2A workers for COVID-19. Based on the testing results of these 1195 H-2A workers, the proportion infected with SARS-CoV-2 was low. The pilot’s low positivity proportion correlated with a 2020 report on the low incidence of COVID-19 among Mexican residents. During the time of the pilot, approximately 3.0% of Mexican residents tested positive for SARS-CoV-2 since the pandemic began [8]. Mexico was the country of focus because most H-2A visas are issued to Mexican nationals, and the panel physician testing sites are in two of the largest H-2A visa-issuing cities in Mexico: Monterrey and Tijuana [3, 9].

Both testing locations (Monterrey and Tijuana) displayed reasonable turnaround times (sample collection to result reported). However, when stratified by test location, Monterrey had a longer overall turnaround time than Tijuana, although still very reasonable given the higher volume of testing completed in Monterrey. These turnaround times were critical for the employers’ next steps. Since COVID-19 testing was voluntary for the employers, reporting of the results needed to be quick and efficient to encourage participation. The main goal of the employers was to recruit H-2A workers as quickly as possible to fill positions in the United States. If turnaround times were longer than expected, the employers might decide that the benefits of COVID-19 testing for their workers were not worth the loss of potential time that the H-2A workers could be working and supporting their companies. Monterey’s high volume of COVID-19 testing corresponds with a report indicating two-thirds of H-2A visa issuances happened in Monterrey during March 2020 [9].

Historically, panel physicians have shown strong expertise in the pre-departure examination of migrants [10]. Therefore, the use of panel physicians for this creative testing strategy proved advantageous for providing COVID-19 testing services in Mexico. Panel physicians were not only meeting but exceeding the industry standard diagnostic test result turnaround time of 1 to 2 days [11]. The testing and subsequent isolation of positive cases prevented potential outbreaks on farms and interruptions of harvest patterns by protecting H-2A workers from COVID-19 before, during, and after migration to the US. The model used in this pilot should be considered for future potential service delivery, such as vaccine administration.

H-2A workers’ migration depends on several factors: crops, harvest patterns, seasonal work needs, etc. [1]. Therefore, their first intended US location was collected to visualize their migration pattern. Although we do not have data on migration patterns to understand how generalizable our results are, the states to which these H-2A workers migrated included some of the top agricultural states in the United States: Florida, Georgia, California, and North Carolina [12].

This project was the only effort, to date, that has been made to provide COVID-19 testing to H-2A workers before entry to the US. This pilot was creative and significant because H-2A workers are an essential workforce. They are also identified as a vulnerable population since they may not be able to fully protect themselves against infection when making decisions on transportation, housing conditions, and labor conditions [13].

Because H-2A workers are an essential asset to the US food supply, many employers took responsibility for protecting their H-2A workers by signing them up for testing in this pilot. In a recent study, H-2A workers reported being reluctant to undergo COVID-19 testing because they feared that a positive result would impact their ability to work [13]. Testing workers in Mexico, before migrating to the United States helped employers make informed decisions for worker safety and provided workers with a sense of security by eliminating uncertainty. It also prevented delaying migration and productivity. For a given employer, this strategy may protect their entire cohort of workers, as it identifies and isolates infected individuals before they can expose others during the sometimes-crowded conditions associated with travel and post-arrival housing arrangements.

One of the main limitations of this project is the small sample size of 1195 H-2A workers which represents 0.46% out of an estimated 258,000 H-2A visas issued in the fiscal year 2021 [14]. Another limitation observed was outreach and advertising, which had a major effect on the population tested. Although CDC worked with NCAE and other entities to advertise the pilot, a large majority of the H-2A workers that were tested came from one contracting agency. If more employment companies had participated in this pilot, it might have provided additional opportunities to make the data more generalizable. Unfortunately, no data is available to compare H-2A origins in the overall H-2A program. Also, having the sample come from one main agent limits the ability to distinguish how many employers or farms are represented in that subgroup.

Collecting more granular coordinates for mapping H-2A workers’ current residences could be done for future analysis and result in a more accurate tracking of H-2A workers’ migration. Many of the H-2A workers tested resided in small, rural towns in Mexico and only state-level mapping was performed for this pilot. Future investigation of more granular coordinates could prove useful in assessing the finer-level migration of H-2A workers throughout the United States.

This pilot was conceived and organized before COVID-19 vaccine availability. However, vaccines were starting to become available once testing began. For this reason, we could not incorporate the COVID-19 vaccine into the pilot or assess worker, employer, or agent perspectives on the COVID-19 vaccination. Following the pilot, NCAE and many employers have inquired about H-2A workers being vaccinated through this public health response screening pilot. At present, COVID-19 vaccinations may only be provided in Mexico at Ministry of Health clinics.

Future efforts to protect the H-2A workforce can prioritize obtaining COVID-19 vaccination in Mexico’ or something similar. The White House Proclamation, at the time of the pilot, regarding COVID-19 vaccination before entering the United States did not apply to H-2A workers because they are designated as essential workers [15, 16]. If panel physicians can provide COVID-19 vaccinations, the results of this pilot suggest that using a panel site to vaccinate H-2A workers can be part of a strategy to protect this essential workforce.

In summary, as the pilot was being presented and advertised, employers reached out eagerly to take advantage of this opportunity to get their H-2A workers tested for COVID-19. The turnaround times met and exceeded industry standards, which gave employers strategic information, in a timely fashion, to make decisions related to H-2A workers’ safety. The testing of H-2A workers in Mexico gave employers strategic information to help keep their employees safe and helped protect this workforce. The pilot demonstrated the feasibility of using panel physicians to provide COVID-19 services to H-2A agricultural workers pre-departure. For future programs, employers revealed great interest in using this model for providing COVID-19 vaccinations.
